# Parameter Identification in a Generalized Time-Harmonic Rayleigh Damping Model for Elastography

**DOI:** 10.1371/journal.pone.0093080

**Published:** 2014-04-01

**Authors:** Elijah E. W. Van Houten

**Affiliations:** Département de génie mécanique, Université de Sherbrooke, Sherbrooke, QC, Canada; Rensselaer Polytechnic Institute, United States of America

## Abstract

The identifiability of the two damping components of a Generalized Rayleigh Damping model is investigated through analysis of the continuum equilibrium equations as well as a simple spring-mass system. Generalized Rayleigh Damping provides a more diversified attenuation model than pure Viscoelasticity, with two parameters to describe attenuation effects and account for the complex damping behavior found in biological tissue. For heterogeneous Rayleigh Damped materials, there is no equivalent Viscoelastic system to describe the observed motions. For homogeneous systems, the inverse problem to determine the two Rayleigh Damping components is seen to be uniquely posed, in the sense that the inverse matrix for parameter identification is full rank, with certain conditions: when either multi-frequency data is available or when both shear and dilatational wave propagation is taken into account. For the multi-frequency case, the frequency dependency of the elastic parameters adds a level of complexity to the reconstruction problem that must be addressed for reasonable solutions. For the dilatational wave case, the accuracy of compressional wave measurement in fluid saturated soft tissues becomes an issue for qualitative parameter identification. These issues can be addressed with reasonable assumptions on the negligible damping levels of dilatational waves in soft tissue. In general, the parameters of a Generalized Rayleigh Damping model are identifiable for the elastography inverse problem, although with more complex conditions than the simpler Viscoelastic damping model. The value of this approach is the additional structural information provided by the Generalized Rayleigh Damping model, which can be linked to tissue composition as well as rheological interpretations.

## Introduction

The importance of damping models in elastography has become clearer in recent years as attenuation levels measured by elastographic imaging have been linked to diseases of the liver [1]–[6] and brain [7]–[13]. A number of methods have been proposed for reconstructing the Viscoelastic (VE) properties of soft tissue [14]–[18], including an iterative, nonlinear inversion method [19], [20]. These methods have all targeted the development of images of the storage (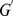) and loss (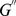) modulus distributions within the tissue in question. Some have gone on to investigate the frequency dependent behavior of these two parameters [5], [11], as well as multi-frequency reconstruction methods to improve the quality of the VE parameters reconstruction across a range of frequencies [18]. While these methods have already demonstrated the important role of tissue attenuation in differentiating tissue type and identifying lesions, linear VE provides a relatively simplified model for understanding the complex, non-linear attenuation observed in *in-vivo* tissue.

Rayleigh Damping (RD), also known as proportional or Caughey damping, is a damping model with origins in numerical structural mechanics and is characterized by providing attenuation effects that are proportional to both elastic and inertial forces. As such, RD is a more diversified damping model than VE, where attenuation forces are related uniquely to elastic forces. From a numerical perspective, RD has the advantage that the damping matrix can be can be modally decomposed using the eigensystem developed from the undamped system, and has been shown to be useful in applications such as an absorbing boundary layer to remove spurious reflections in machine vibration and seismic models [21]. A rheological interpretation of RD can also be developed for weak to moderate damping levels [22].

A Generalized RD formulation has been developed previously for use in elastography imaging [23]. This time-harmonic formulation differs from the traditional RD configuration in the sense that damping effects are developed through complex valued shear modulus (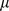) and density (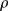) parameters, where the imaginary parts of 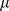 and 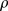 are allowed to vary independently from their real counterparts. This is in contrast with the classical RD structural model, where the damping matrix is composed of scalar combinations of the mass and stiffness matrices. The difference is subtle, but important, as the use of the complex shear modulus value separates damping effects in distortional and dilatational waves, which will be seen to be critical for identifiability of the system. The use of Generalized RD in elastography is of interest mainly due to: a) the simplicity of the model, particularly in Finite Element (FE) formulations; b) the larger range of attenuation behavior the model is able to accommodate, where biological tissue is known to exhibit high levels of complex, non-linear damping; and c) the additional damping parameter provided by the imaging process, which has shown sensitivity to material structure, such as the difference between gel and porous materials as well as cancerous and healthy breast tissue [24].

The goal of this paper is to investigate the conditions in which the parameters for a Generalized RD model can be identified in the elastography problem, based on measured motion data within the specimen. The reason to be concerned with identifiability in the RD case has to do with the simplified form of the classical RD reconstruction problem, which can be written as
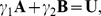
where complex valued 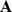 and 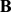 represent the stiffness and mass components of the structure. These parameters are to be identified based on measurements of complex valued 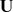. While this case is only identifiable to a linear relation between 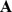 and 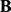,
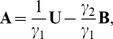
it will be shown that this is in fact an over-simplification of the Generalized RD reconstruction problem that does not account for the dual modes of elastic wave transmission. Additionally, even in the single wave transmission case, it can be shown that the addition of multi-frequency information is sufficient to make the Generalized RD inverse problem uniquely posed.

## Analysis and Methods

### Generalized Time-Harmonic Rayleigh Damping

Classical RD is a structural mechanics formulation based on the discretized dynamic model
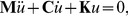
(1)with mass and stiffness matrices, 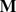 and 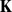, and a damping matrix, 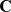, of the form
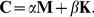
(2)


In contrast, the *Generalized* RD formulation considered in this work is a continuum interpretation of discretized classical RD, with damping effects proportional to shear and inertial forces. In the case of time-harmonic displacements, where 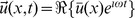 for the complex valued 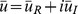, the damping coefficients in Eq. 2 can be defined in terms of complex valued density, 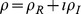, and shear modulus, 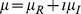, where [23]

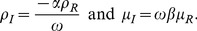
(3)


We note here that the complex valued shear modulus components are directly related to the storage and loss modulus, with 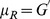 and 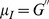. The damping ratio, 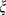, for an RD system is then given by [25],
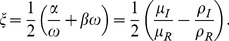
(4)


The elastography problem in a Generalized RD system is then to reconstruct the distribution of the elastic properties 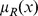, 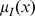 and 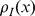 from a measured displacement field, 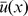. In general, for soft tissue elastography, the mass density, 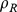, can be assumed *a priori* as equal to that of water, *i.e.* 1000 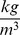. As a point of comparison, the time-harmonic VE model provides damping effects due to a single elastic property, 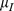, such that the elastography problem in a purely VE system is to reconstruct 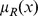 and 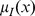 from a measured displacement field, 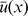.

#### Defining the Generalized Rayleigh Damping Parameters

A physical interpretation of the Generalized RD parameters can be developed by considering the elastic equilibrium of the time-harmonic system through Navier's equation, written as
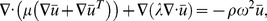
(5)with complex valued 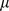 and 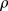 defined as above in Eq. 3. In general, for soft tissue elastography, the system is considered nearly incompressible, with a real valued bulk modulus 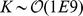 and
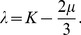
(6)


Dilatational attenuation can be considered, where 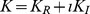, however the levels of damping in this compressional wave propagation will be negligible for higher frequencies where poroelastic effects are minimal [26].

Eq. 5 can be rewritten in the form of an undamped elastic operator 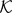, a damping operator 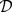 and an inertial operator 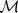, all acting on the time harmonic displacements, 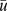. This gives
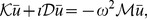
(7)with
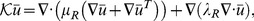
(8)




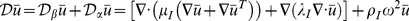
(9)and
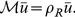
(10)


Substitution of 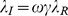 and Eq. 3 into Eq. 9 and defining the time-harmonic velocity as 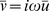, gives the viscoelastic damping operator,
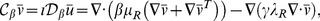
(11)while substitution of Eq. 3 into Eq. 9 gives an inertial damping operator,
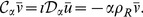
(12)


From Eqs. 11 & 12, the interpretation of the Generalized RD damping operator, 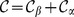, is relatively straightforward. The component 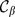 represents traditional viscoelastic damping, where attenuation is related to strain rate. The component 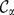 provides a second damping term directly proportional to local velocity, as if the solid matrix were moving through a stationary viscous fluid. In the case of medical imaging, both of these components have a meaningful mechanical analogy to soft tissue structure.

This Generalized RD formulation can be compared with the classical RD formulation by considering the case where 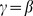, 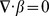 and 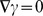, so that
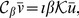
(13)and
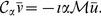
(14)We note that, in this case, Eq. 7 becomes
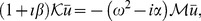
(15)which, as a single complex valued equation, cannot be solved for two independent parameters 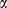 and 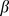. However, the conditions which lead to this case, notably the requirement that 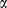 and 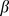 be essentially homogeneous, are not expected in *in-vivo* tissue, and Eq. 6 is commonly used to define 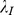, so that Eq. 9 becomes
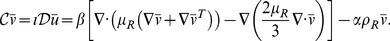
(16)


The implications of this particular case on the identifiability of the RD parameters will be seen in the following sections.

### Uniqueness of the Generalized Rayleigh Damping System

A first step in analyzing the identifiability of the RD parameters, 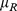, 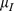 & 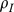, is to consider the existence of a VE system equivalent to the RD system but with a strictly real valued density of 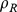. If such a system exists, the RD parameters needed to define a particular motion field, 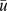, are not unique, and thus they cannot be identified without additional information. To develop the equivalence condition, we consider alternative operators for Eq. 7, so that we have
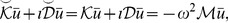
(17)where
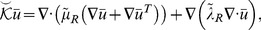
(18)and
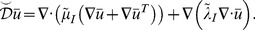
(19)


The form of 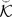 and 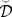 can be determined by first moving 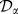 to the right-hand-side of Eq. 7, to obtain
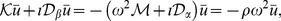
(20)and then multiplying both sides by by 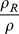, which, after expansion of the derivative terms and combining 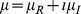 and 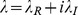, gives
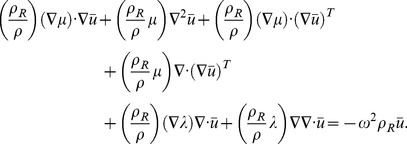
(21)


Making use of the product rule formulation 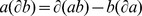, as well as *effective* moduli 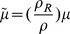 and 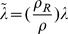 [24], Eq. 21 can be rewritten as
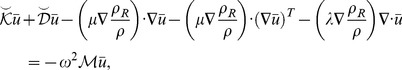
(22)with the operators defined in Eqs. 18 & 19. We see from Eq. 22 that Eq. 17 only holds for the case when the spatial derivative 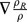 is zero, *i.e.* when 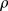 is homogeneous or when 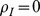. In the case of heterogeneous density or 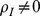, Eq. 22 indicates that some evidence of the intertial RD operator, 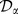, will be present in the motion field, 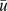. We note that
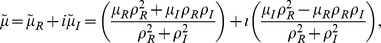
and
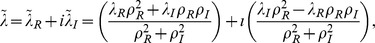
such that, even in the case where 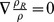, the condition 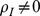 ensures that 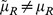, 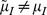, 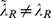 and 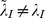. The presence of 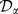 in the system that generates 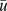 will thus effect measurements of both shear stiffness and viscosity made with a damping operator in the form of 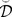.

### Conclusions from Analysis of the Generalized System

Eq. 22 shows that a direct VE equivalent to an RD system is only possible when 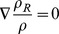. This is an important result as, in general, the material property distributions observed within soft-tissue will be highly heterogeneous, and thus the RD and VE systems are, in general, not equivalent. The task still remains to correctly identify the parameters of the RD system based on the measured motions. Eq. 15 has already indicated that this is generally possible so long as the tissue is heterogeneous and 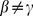, *i.e.* dilatational wave attenuation is not at the same level as shear wave attenuation. The exact conditions under which parameter identification is possible are analyzed in the following section.

### A Spring-Mass Analogy

**Figure 1:**
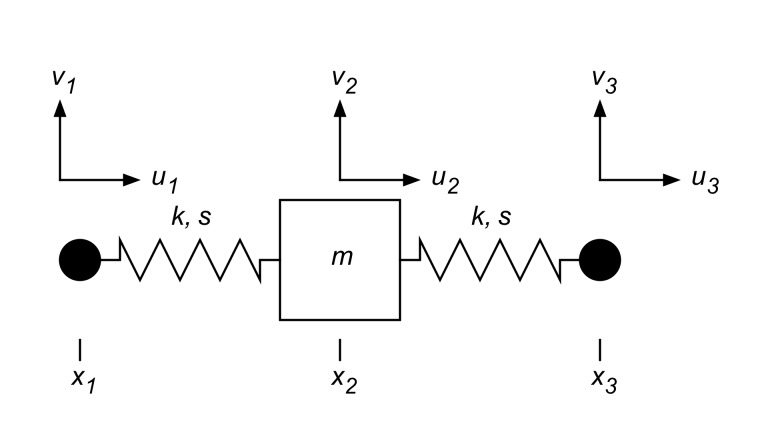
A three mass spring-system as an elastography analogy. To eliminate the need for known forcing information in the elastography analysis, a three mass system is analyzed, where the displacements of masses 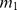 and 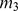 are considered measured data and used to calculate system parameters.

To explore the concept of parameter identification in Generalized RD elastography more closely, we start by considering a simple, locally homogeneous spring-mass system, where the spring stiffness, 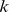, has a VE component, 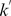, while the mass, 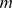, has an inertial damping component, 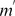. To focus on the case of *elastography* imaging, the spring system is constructed of three masses, connected by two springs, all in series, as shown in Figure 1. The reason for this arrangement is that typically, in elastography imaging, the applied forces, 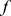, are unknown, and therefore cannot be called upon for the *inverse* problem of solving for the elastic parameters given the motions. Instead, only motion data is obtained, so in the two cases presented here we will consider the *data* as the *axial* displacements of the system, 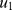, 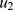 and 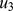, measured at positions 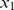, 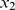 and 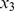. In addition, we will see that supplementary data can be obtained from measuring the corresponding *transverse* displacements, 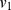, 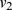 and 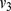. Without forcing information, the trivial solution, 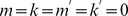, is a possibility for the general inverse problem. To eliminate this solution, elastography methods typically assume the mass, 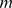, is a known quantity. In soft-tissue imaging, this corresponds to the assumption that biological soft tissue has a density equal to that of water.

A note on noise. In general, direct elastography inversion schemes such as those shown here are highly unstable, and the condition of the inversion matrix, 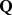, deteriorates rapidly with the presence of noise in the measured displacements. However, the purpose of this work is *not* to consider the impact of noise on the elastographic inverse problem. Any suitable approach to elastography imaging will have addressed the issue of the poor condition of the inverse solution matrix, and a number of well documented methods for resolving these issues have been presented in the literature. The choice of the direct inversion approach is made here because it allows explicit inspection of the inversion matrix to determine if the inverse problem is full rank. The sensitivity of these problems to noise in the data means that even if the inverse matrix is full rank, practical solutions from measured data requires filtering and regularization, despite the problem being ''well posed''.

### Case I: A 1D, Homogeneous System

We start with the simplest of all cases, with a single mass, 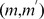, supported in series between two homogeneous springs, 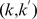, with steady displacements applied at both ends, 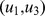, at radial frequency 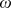.

#### Forward Problem

First, we consider the equation of motion for the interior mass at 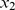, 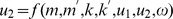. This is obtained from considering the equilibrium equation for the system, given by
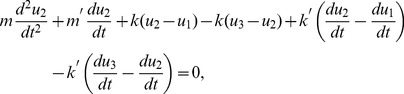
(23)which, with a time-harmonic assumption that the displacement will have the form 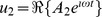, 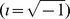, leads to the equilibrium form
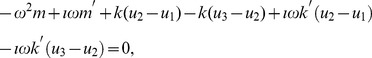
(24)with the solution
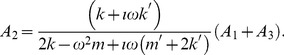
(25)


Eq. 25 can be rewritten in the form
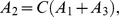
where 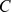 has real and imaginary parts
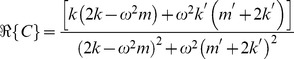
(26)and
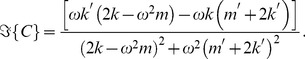
(27)


#### Inverse Problem

The inverse problem in this case is ill posed, as can be deduced from the fact that the relationship between 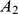 and the *driving* conditions, 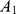 and 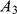, is only governed by two numbers, 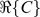 and 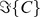. If we assume 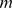 is known, that leaves 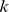, 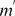 and 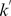 to determine, with only two equations to use! The *direct* inverse problem can be written as
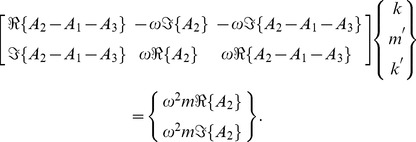
(28)


The inverse solution matrix in this case, 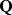, is clearly only rank 2, meaning we can compose the solution of any two parameters as functions of the remaining two. For example:
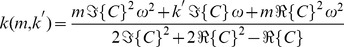
(29)and
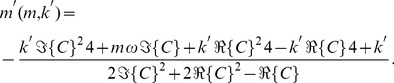
(30)


#### Conclusions

The inverse problem in this case is not uniquely posed and the direct inversion matrix is rank deficient. There is no way to calculate a unique value of 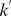 and 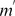 given a single displacement measurement in a single dimension. We could consider adding an additional mass to the system to obtain more information, essentially adding another instance of Eq. 25, in the form
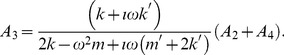
(31)


However, the additional measurement of 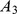 provides no new information, as
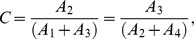
and the expanded inversion matrix, 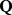, is still rank 2, with
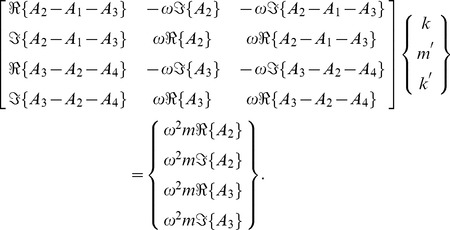
(32)


### Case II: A 1D System, with Multiple Frequencies

The detection of the individual RD components requires additional, independent information in order to be uniquely posed. One option for obtaining this information is to consider measurements at different excitation frequencies, say 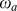 and 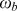, such that
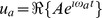
and
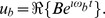
The inverse problem system then becomes
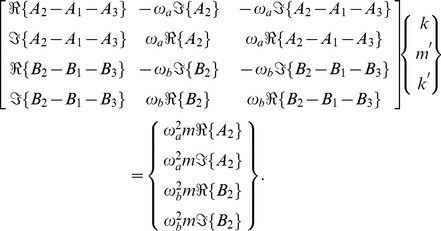
(33)


We can examine the rank of this system by considering the determinant of the submatrix 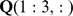, whose numerator, for the case where 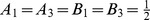, is given by
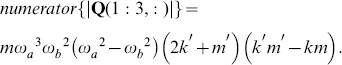
(34)


Eq. 34 has two real valued roots, 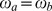 and 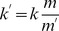 (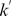 and 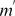 are positive reals, so the factor 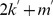 will never be zero). The first of these roots reduces the system to the single mass, single frequency case described above. All additional information is identical to the original data, and the inversion matrix 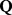 is once again rank 2. The importance of the second root is seen in considering the determinant of the submatrix 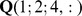, which has the numerator
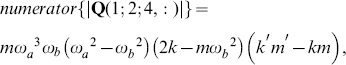
(35)where we see the common roots at 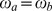 and 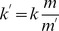. Both of these conditions will thus reduce the inversion matrix to rank 2. The third root of 35, 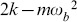, corresponds to undamped resonance at 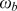, but due to the fact that this root does not appear in Eq. 34, this condition does not reduce the inverse problem matrix to rank 2.

#### Conclusions

From Eqs. 34 & 35 we see that the multi-frequency inverse problem for RD systems is generally uniquely posed, and, except in the case where 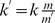, Eq. 33 allows the determination of the two independent damping components 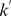 and 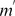 from data taken at two distinct frequencies. In practice, we note that material properties will often change with frequency, meaning that 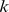, 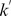, and even 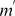 should be expected to be different at frequencies 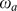 and 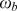, thus making the inverse problem more complex than that posed in Eq. 33. This issue can be addressed by developing simple frequency dependency relationships for 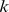, 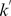, and even 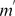, such as the power law relation, 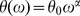, and then adding additional frequency data, *i.e.* 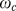, to account for the additional parameters to be determined, 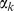, 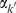 and 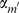.

### Case III: A 2D System

The frequency dependency of mechanical properties can be avoided in RD parameter reconstruction by considering a single frequency 2D wave propagation model for the spring-mass system shown in Figure 1. By allowing the mass to vibrate both axially, with displacement 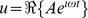, and transversely, with displacement 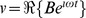, the model allows the propagation of both shear and longitudinal waves. From linear elastic theory, propagation of shear and longitudinal waves is governed by the elastic ''moduli'' 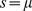 and 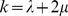, respectively. For soft tissue imaging, it is common to make use of the fact that tissue is nearly incompressible due to its high water content. Thus, the bulk modulus, 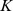, of the tissue can be specified at a very high numerical value (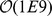). The value of 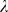 can then be calculated from the relation
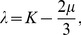
(36)which leads to the longitudinal wave ''modulus''
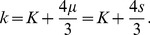
(37)


To determine the VE component of 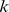, it is assumed that 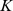 is real valued, so that 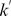 is determined by substituting the imaginary shear modulus component, 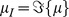, into Eq. 37. We'll consider the case of complex valued 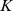 shortly. Thus, we have for the longitudinal wave attenuation
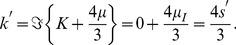
(38)


#### Forward Problem

With the time-harmonic assumption noted above and the effective longitudinal wave modulus and attenuation given by Eqs. 37 & 38, we have the equilibrium conditions
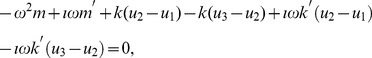
(39)and
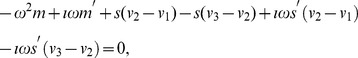
(40)with the solutions
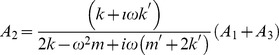
(41)and
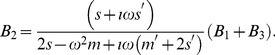
(42)


#### Inverse Problem

The inverse problem system then becomes
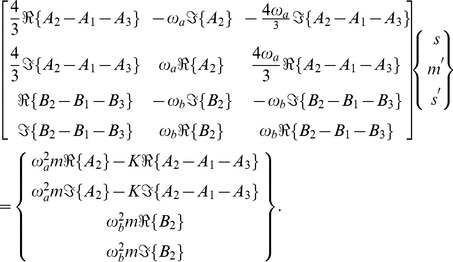
(43)


We can examine the rank of this system by considering the determinant of the submatrix 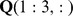, whose numerator, for the case where 
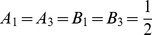
, is given by
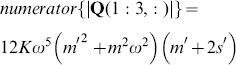
(44)


Eq. 44 has no real roots, given that 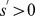 and 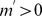 for RD systems. We note also that the determinant of the submatrix 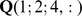 has the numerator
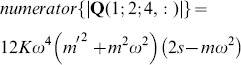
(45)with a root at 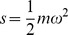, which corresponds to the undamped resonance case in the shear displacements, 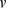. However, as this root only appears in Eq. 45, this condition does not reduce the inverse problem matrix to rank 2.

#### Conclusions

From Eqs. 44 & 45 we see that the 2D problem for RD systems is generally uniquely posed, and Eq. 43 allows the determination of the two independent damping components 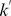 and 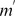 from shear and longitudinal wave data.

### Case IV: A 2D System, with Relative Damping Components

The 2D problem above can be generalized for a system where attenuation occurs in both dilatational and distortional wave propagation. In this case, the shear and longitudinal stiffnesses, 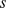 and 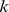, as well as their VE components, 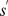 and 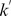, can be directly related by factors 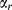 and 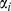, such that
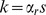



and
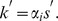



#### Forward Problem

With the time-harmonic assumption, we have the equilibrium conditions
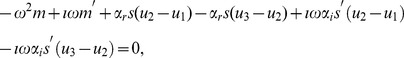
(46)and
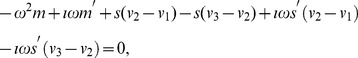
(47)with the solutions
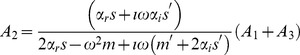
(48)and
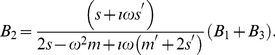
(49)


#### Inverse Problem

The inverse problem system then becomes
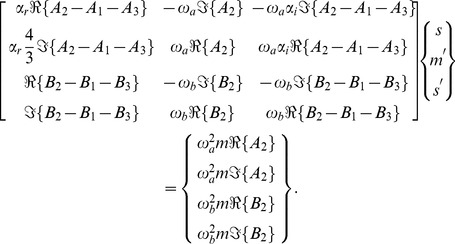
(50)


We can examine the rank of this system by considering the determinant of the submatrices 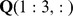 and 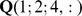, whose numerators, for the case where 
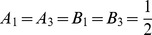
, are given by
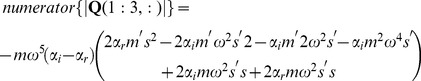
(51)and
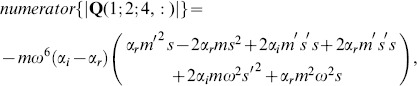
(52)respectively. We note that both numerators share the factor 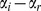, meaning that the inverse problem becomes ill posed in the case where 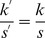.

#### Conclusions

From Eqs. 51 & 52 we see that the 2D problem for RD systems is generally uniquely posed, except for the case where the damping ratios for distortional and dilatational wave attenuation are equal, *i.e.* whenever
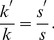
(53)


Equivalent to Eq. 4, the damping ratio, 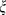, for wave propagation in a mode governed by stiffness 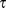 is given by the relation
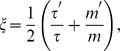
where 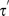 is the VE attenuation in the given propagation mode (*i.e.* either 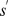 for shear waves or 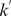 for longitudinal waves). In this case, we see that the condition for which the inverse problem becomes ill posed, given by Eq. 53, is equivalent to the damping ratio for wave attenuation for the shear and longitudinal waves being equal. In this case, no new information is added to our inverse problem system by considering the two different wave propagation cases, and the problem becomes rank deficient.

### Case V: A 2D System, with Dilatational Damping

The generalized case discussed above breaks down in the case where 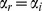. In the case of damped dilatational waves, the coefficients 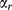 & 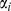 are defined as
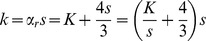
and
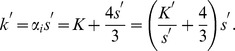
The condition 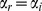 is then equivalent to
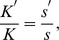

*i.e.* the condition of equivalent damping ratios for distortional and dilatational wave propagation. This singularity condition can be eliminated by considering 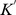 as a separate unknown in the RD inverse problem (we can maintain the ''nearly incompressible'' condition by assuming 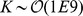).

#### Forward Problem

With the time-harmonic assumption, we have the equilibrium conditions
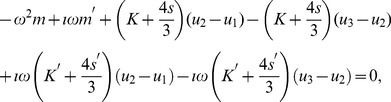
(54)and
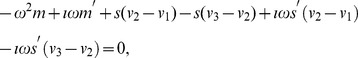
(55)with the solutions
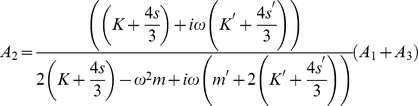
(56)and
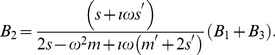
(57)


#### Inverse Problem

The inverse problem system then becomes
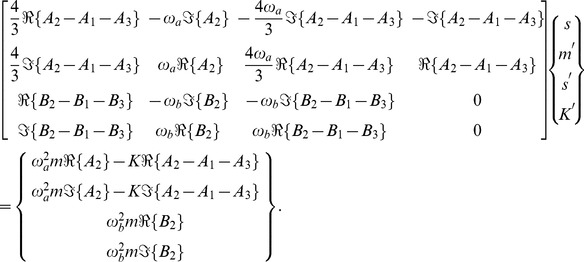
(58)


We can examine the rank of this system by considering the determinant of the full matrix 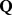, whose numerator, for the case where 
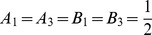
, is given by
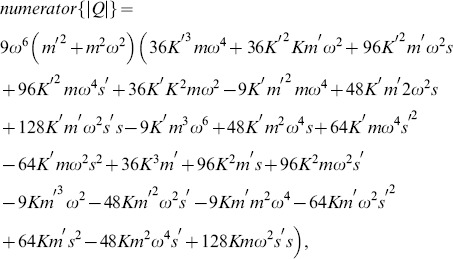
(59)whose roots, given as values of 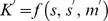, are complex enough to essentially eliminate the chance of them occurring for reasonable values of 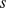, 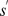 & 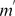 in soft tissue.

#### Conclusions

Eq. 59 indicates that the general RD inverse problem, even in cases with dilatational wave attenuation, is generally uniquely posed once we include 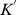 among the parameters for reconstruction. While an interesting theoretical result, the practicality of obtaining meaningful results from dilatation measurements in Elastography is severely limited, given their long wavelengths and the the susceptibility of these measurements to noise.

### Conclusions from Spring-Mass Analysis

A summary of the conclusions from the above cases is given below:

- From Case I we see that the simple problem of deducing from 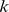, 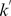 and 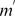 from the 1D displacement of a single mass at a single frequency is impossible. This is not a surprise, as the *data* here consists of a single complex number, *i.e.* two measurements cannot produce three parameters. The inverse problem matrix, 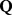, is rank 2.
- From Case II we see that, in general, the addition of data taken at another frequency makes the RD inversion problem uniquely posed. The inverse problem matrix is rank 3 except in the special case where 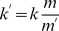, which corresponds to a damping ratio 
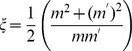
.
- From Case III, and more generally Case IV, we see that the addition of shear wave data from the transverse direction makes the problem uniquely posed, so long as the attenuation differs between the shear and longitudinal wave propagation.
- From Case V, we see that the general problem of RD parameter reconstruction becomes uniquely posed if we include the dilatational attenuation, 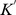, as an unknown parameter, even in the case where 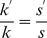

We see that the spring-mass RD reconstruction problem is generally uniquely posed with additional measurements, either from the propagation of waves along a different mode, or from additional frequency information. In practice, these two information sources contain their own challenges. In the case of measurements at additional frequencies, the frequency dependency of the parameters comes into play, and in itself requires additional information (as well as good models for this frequency dependency) in order to pose a reasonable reconstruction problem. In the case of deducing information from longitudinal waves in elastography, the problem arises from the *nearly incompressible* nature of soft tissue, where the longitudinal wavelength becomes very long 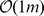 in comparison to the size of the objects being measured 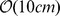. Accurately characterizing these wavelengths within the medium is thus highly susceptible to noise.

## Discussion

Overall, we can say that the RD parameters are identifiable, given certain conditions and assumptions on the damping behavior of the elastic material. To start with, in a region of heterogenous material properties, where the term 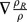 is unlikely to disappear, analysis of the generalized RD system shows that it has no purely VE equivalent, and thus there is a valid reason to consider RD reconstruction. Next, to determine the RD parameters, the assumptions required for identification are not particularly onerous, specifically, the idea that the attenuation of dilatational waves is of a different order to the attenuation of shear waves is quite reasonable and easy to justify. The real issue, however, is the dependence of the reconstruction on measurements of the dilatational wave component itself, which are easily corrupted by the presence of noise. One way to alleviate this problem is by introducing multiple frequency data into the reconstruction problem, which, except in special circumstances, renders the RD identification problem uniquely posed. Multi-frequency reconstruction introduces its own set of complications however, due to the frequency dependence of the parameters in the elastic equilibrium equations. In short, RD parameter identification is possible, but not a simple affair. The intrigued reader asking “why bother?” is encouraged to consult the results presented in [24] for some evidence of the potential value of RD parameter data in elastography imaging.
